# PTK6 Regulates IGF-1-Induced Anchorage-Independent Survival

**DOI:** 10.1371/journal.pone.0011729

**Published:** 2010-07-23

**Authors:** Hanna Y. Irie, Yashaswi Shrestha, Laura M. Selfors, Fabianne Frye, Naoko Iida, Zhigang Wang, Lihua Zou, Jun Yao, Yiling Lu, Charles B. Epstein, Sridaran Natesan, Andrea L. Richardson, Kornelia Polyak, Gordon B. Mills, William C. Hahn, Joan S. Brugge

**Affiliations:** 1 Department of Cell Biology, Harvard Medical School, Boston, Massachusetts, United States of America; 2 Department of Medical Oncology, Dana-Farber Cancer Institute, Boston, Massachusetts, United States of America; 3 Department of Pathology, Brigham and Women's Hospital, Boston, Massachusetts, United States of America; 4 Department of Systems Biology, University of Texas MD Anderson Cancer Center, Houston, Texas, United States of America; 5 Sanofi-Aventis, Cambridge, Massachusetts, United States of America; 6 Broad Institute of Harvard and Massachusetts Institute of Technology (MIT), Cambridge, Massachusetts, United States of America; Dresden University of Technology, Germany

## Abstract

**Background:**

Proteins that are required for anchorage-independent survival of tumor cells represent attractive targets for therapeutic intervention since this property is believed to be critical for survival of tumor cells displaced from their natural niches. Anchorage-independent survival is induced by growth factor receptor hyperactivation in many cell types. We aimed to identify molecules that critically regulate IGF-1-induced anchorage-independent survival.

**Methods and Results:**

We conducted a high-throughput siRNA screen and identified PTK6 as a critical component of IGF-1 receptor (IGF-1R)-induced anchorage-independent survival of mammary epithelial cells. PTK6 downregulation induces apoptosis of breast and ovarian cancer cells deprived of matrix attachment, whereas its overexpression enhances survival. Reverse-phase protein arrays and subsequent analyses revealed that PTK6 forms a complex with IGF-1R and the adaptor protein IRS-1, and modulates anchorage-independent survival by regulating IGF-1R expression and phosphorylation. PTK6 is highly expressed not only in the previously reported Her2^+^ breast cancer subtype, but also in high grade ER^+^, Luminal B tumors and high expression is associated with adverse outcomes.

**Conclusions:**

These findings highlight PTK6 as a critical regulator of anchorage-independent survival of breast and ovarian tumor cells via modulation of IGF-1 receptor signaling, thus supporting PTK6 as a potential therapeutic target for multiple tumor types. The combined genomic and proteomic approaches in this report provide an effective strategy for identifying oncogenes and their mechanism of action.

## Introduction

Adhesion to extracellular matrix (ECM) provides epithelial cells with critical cues about their environment that are required for their proliferation, survival and tissue organization. Loss of attachment to matrix compromises viability of normal epithelial cells through a variety of mechanisms that help preserve tissue homeostasis and prevent aberrant growth (reviewed in [1], [2]). Detachment from matrix triggers apoptosis, termed anoikis, via both intrinsic and extrinsic death pathways. However, most tumor cells have acquired the ability to resist anoikis and this property is believed to be critical for tumor cell dissemination and survival in altered matrix environments [1], [2]. Genes that have been demonstrated to suppress anoikis also promote metastases in vivo, further supporting a critical role for anoikis regulation in tumorigenesis [3], [4].

Tumor cells adopt several different strategies to evade anoikis including: (1) activation of survival pathways such as those regulated by Erk/MAPK and Akt through oncogenic mutations or constitutive growth factor receptor activation; (2) modulation of expression or activity of anti-apoptotic and pro-apoptotic proteins including Bcl2 family members, and (3) altered expression and engagement of integrins by basement membrane proteins produced via autocrine mechanisms (reviewed in [1], [2]). Large-scale cDNA screens have identified several candidate genes that when overexpressed either alone or in combination with other oncogenes suppress anoikis through any or all of these mechanisms [4].

As a complement to these gain-of-function screens, loss-of-function screens also provide insight into mechanisms that are necessary for anoikis suppression and identify potential targets for therapeutic intervention. Screens utilizing small molecule inhibitors have previously been reported [5], [6]; these studies have highlighted multiple ways in which anoikis resistance may be overcome, including manipulation of the extrinsic cell death pathway and hypoosmotic stress. Here we present a novel siRNA screen designed to identify regulators of IGF-1 receptor (IGF-1R)-driven anoikis resistance of breast epithelial cells.

IGF-1R has been shown to be expressed in the majority of human breast cancers with evidence of sporadic amplification in a small proportion of these cases [7], [8]. Although initially thought to correlate with estrogen receptor (ER) expression, IGF-1R has recently been implicated in multiple breast cancer subtypes and its expression correlates with poor prognoses [9], [10]. IGF-1 stimulation directly induces anoikis resistance of several different epithelial cell types (including breast, prostate, and colon) by activating downstream signaling molecules such as Ras/MAPK and PI3K/Akt [11]. IGF-1R is also required for transformation and anoikis suppression induced by other oncogenes, such as Ras, c-Src, SV40 Large T antigen and the chimeric ETV6-NTRK3 (reviewed in [12], [13]). Based on these findings, we utilized mammary epithelial cells expressing elevated levels of IGF-1R for a siRNA screen to identify mediators of anoikis protection. In addition, use of cells in which a known gene drives protection from anoikis greatly facilitates mechanistic follow-up studies. This siRNA screen led to the identification of PTK6, a member of the Src family of tyrosine kinases that is frequently overexpressed in a variety of tumor types [14]–[17]. Here we demonstrate a critical role for PTK6 in anchorage-independent survival of specific subtypes of breast cancer cells. We also present evidence for IGF-1 receptor regulation as a novel mechanism for survival regulation by PTK6.

## Results

### Loss-of-function screen to identify genes involved in anchorage-independent survival induced by IGF-1R stimulation

**Figure 1:**
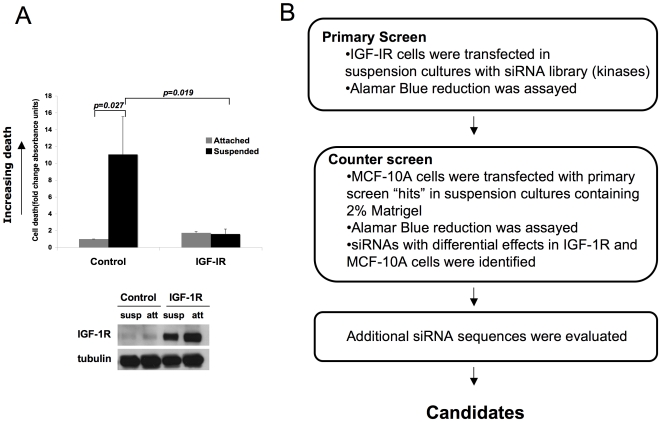
siRNA screen identifies regulators of IGF-1-induced anchorage-independent survival. A) IGF-1R hyperstimulation enhances anchorage-independent survival of MCF-10A cells. MCF-10A cells overexpressing vector control or IGF-1R (IGF-1R) were cultured on adherent plates or in suspension cultures for 48 hours. The cells were collected and cell death was assessed by measuring cytoplasmic histone and DNA content in triplicate wells (Roche Diagnostics). Higher values indicate more death. The fold change is the average of three independent experiments. Error bars indicate standard deviation. Western blot analysis shows relative IGF-1R levels in the cell lines. B) Candidate siRNAs that are critical for IGF-1R-induced anchorage-independent survival were identified by a loss-of-function screen. In the primary screen, a library of siRNA oligonucleotides targeting all human kinases was screened using the IGF1R overexpressing MCF10A line. Alamar blue reduction was assayed. Positives from the primary screen were counter-screened in MCF10A cells cultured in 2% Matrigel in suspension. The screen positives that showed a differential effect in IGF1R-overexpressing cells compared to the MCF10A cells in Matrigel were selected for validation using additional sequences.

In contrast to non-transformed MCF-10A mammary epithelial cells, MCF-10A cells overexpressing IGF-1 receptor (IGF-1R cells) exhibit enhanced survival in suspension cultures with IGF-1 stimulation (Figure 1A). To identify genes that are required for IGF-1 induced anchorage-independent survival, IGF-1R cells were transfected in suspension cultures with a library of individual siRNA oligonucleotides targeting all human kinases and related proteins (Figure 1B). The ability of individual siRNAs to modulate or reverse IGF-1dependent survival was initially assessed by monitoring changes in Alamar Blue reduction signal. Candidate siRNAs identified in the primary screen were rescreened using parental MCF-10A cells cultured in suspension with reconstituted basement membrane proteins to prioritize those siRNAs that more specifically inhibited IGF-1 driven survival. Lastly we screened additional siRNAs targeting the IGF-1R specific hits to identify those genes that were targeted by three or more distinct siRNA sequences.

### PTK6 modulates IGF-1 dependent anoikis resistance

**Figure 2:**
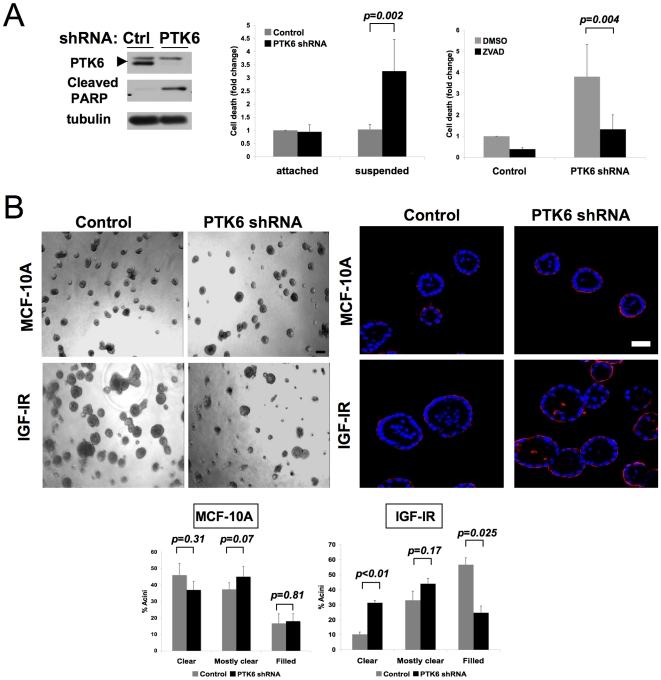
PTK6 downregulation reverses IGF-1-induced anchorage-independent survival. A) An shRNA vector targeting PTK6 reverses IGF-1R-induced anchorage-independent survival. (Left) Western analysis confirmed downregulation of PTK6 expression and demonstrated increased PARP cleavage with PTK6 downregulation in IGF-1R cells in suspension cultures. (Middle) IGF-1R cells expressing control or PTK6 shRNA vector were cultured as attached cells or in suspension cultures in the presence of IGF-1. Cell death was assessed after 48 hours as in Figure 1. The average fold change from three independent experiments is shown. Error bars indicate standard deviation. (Right) PTK6 regulates apoptosis of matrix-detached IGF-1R cells. IGF-1-stimulated IGF-1R cells expressing control or PTK6 shRNA vector were grown in suspension cultures for 48 hours in the presence or absence of ZVAD-fmk (25 µM). Cell death was assessed as in Figure 1. The average fold change from three independent experiments is shown. Error bars indicate standard deviation. B) PTK6 downregulation inhibits morphological changes induced by IGF-1R hyperstimulation. (Left) IGF-1R or parental MCF-10A cells expressing control or PTK6shRNA vector were grown in 3D Matrigel™ cultures for 8 days. Representative phase contrast images are shown. Scale bar indicates 100µm. (Right) PTK6 downregulation inhibits IGF-1R-induced luminal filling. IGF-1R or parental MCF-10A cells expressing control or PTK6 shRNA vector were grown in 3D™ Matrigel cutures for 8 days and stained with anti-laminin 5 (LAMC2) antibody (red) and DAPI (blue). Representative confocal images are shown. Cells in the central lumen were counted and acinar structures were classified as “clear” (no cells in the lumen), “mostly clear” (<5 cells in the lumen) or “filled” (>5 cells in the lumen). The average distribution from three independent experiments is shown. Error bars indicate standard deviation. Scale bar indicates 50 µm.

PTK6 was identified as a “high-confidence” candidate gene because multiple siRNA oligonucleotides caused a significant, preferential inhibition of IGF-1R-driven anchorage-independent viability when compared to parental MCF-10A cells. PTK6 was also considered an attractive candidate for further study because it is highly expressed in multiple tumor types, including breast and ovarian tumors [14]–[16], [18]. Low level expression of PTK6 protein was detected in parental MCF-10A cells grown in suspension cultures; this expression was enhanced in IGF-1R cells (Figure S1A) All three oligonucleotides identified in the phenotypic screen downregulated PTK6 protein expression effectively, as did a pool of siRNA olgonucleotides (OTP) from an independent vendor (Figure S1B). We also identified a lentivirally-delivered shRNA vector that effectively downregulated PTK6 protein expression (Figure 2A, left).

To more specifically determine whether PTK6 downregulation induces cell death of IGF-1R cells in suspension cultures, we utilized Cell Death ELISA assays and monitored cleavage of PARP, a caspase target. Downregulating PTK6 by either transfecting siRNA oligonucleotides or expressing an shRNA vector enhanced death of IGF-1R cells in suspension with enhanced detection of cleaved PARP product (Figure 2A and Figure S1C). The effect of PTK6 downregulation on apoptosis is specific for matrix-deprived cells as downregulation of PTK6 had no effect on attached IGF-1R cells (Figure 2A), supporting the specificity of the screen in identifying genes that modulate survival only in matrix-detached conditions. This death was inhibited by ZVAD-fmk, a pan-caspase inhibitor, further supporting a role for PTK6 in suppressing anoikis induced by matrix detachment (Figure 2A).

### Downregulation of PTK6 inhibits luminal filling induced by IGF-1R hyperstimulation

Acquisition of anchorage-independent survival is believed to play a role in the abnormal accumulation of cells in the lumen of terminal breast acinar structures, a property that is commonly observed in human breast lesions like ductal carcinoma in situ (DCIS). Consistent with this, we have found that filling of the matrix-free lumen of mammary acinar structures in 3D culture models requires induction of both hyperproliferation and anti-apoptotic activity [19].

We and others have previously reported that IGF-1R hyperstimulation of breast epithelial cells results in the formation of abnormally large structures with filled lumen due to hyperproliferation and suppression of death of the centrally localized cells in the presumptive luminal space [20], [21]. To address whether PTK6 plays a critical role in modulating the survival of cells in the luminal space, we examined the effects of PTK6 downregulation on IGF-1-induced acinar formation and luminal filling. PTK6 shRNA expression in IGF-IR/MCF-10A cells suppressed the formation of large, hyperproliferative structures (Figure 2B left). While many PTK6 shRNA-expressing structures resembled parental MCF-10A acini, some were aborted in early stages of outgrowth. These results indicate that PTK6 is also critical for IGF-1R-induced hyperproliferation in 3D culture. In contrast, downregulation of PTK6 had no significant effect on basal morphogenesis of parental MCF-10A cells (Figure 2B left).

We then examined the effect of PTK6 downregulation on IGF-1R-induced luminal filling in 3D cultures. Compared to acini expressing vector control, PTK6 downregulation resulted in a significantly higher percentage of structures with clear lumen (Figure 2B right). Thus, PTK6 is critical for anchorage-independent survival of cells in suspension and in the luminal space of IGF-1R overexpressing acinar structures. The combined effects of PTK6 downregulation on proliferation and anchorage-independent survival contribute to inhibition of abnormal acinar formation induced by IGF-1R stimulation.

### Overexpression of PTK6 enhances anchorage-independent survival

**Figure 3:**
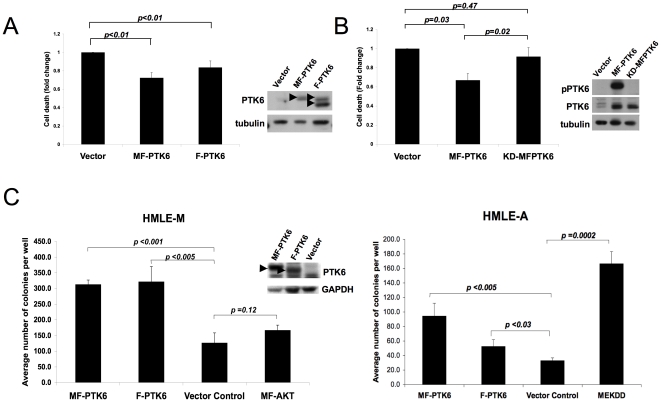
Effects of PTK6 overexpression on anchorage-independent survival. A) Overexpression of PTK6 enhances anchorage-independent survival. MCF-10A cells overexpressing vector control or FLAG-tagged PTK6, with or without a myristylation sequence (MF-PTK6 or F-PTK6, respectively), were cultured in suspension for 48 hours. Cell death was assessed as in Figure 1. The average of three independent experiments is shown. Error bars indicate standard deviation. Western blot analysis shows relative expression levels of PTK6 for each cell line. B) Overexpression of kinase inactive PTK6 does not rescue MCF-10A cells from anoikis. MCF-10A cells overexpressing vector control, MF-PTK6 or a kinase inactive PTK6 (KD-MFPTK6) were cultured in suspension for 48 hours. Cell death was assessed as in Figure 1. The average of three independent experiments is shown. Error bars indicate standard deviation. Relative expression and activity of PTK6 in each cell line was assessed by Western blot analyses. C) Overexpression of PTK6 enhances soft agar growth in collaboration with other oncogenes. HMLE cells expressing MEK^DD^ (HMLE-M) or myristylated Akt (HMLE-A) were engineered to express vector control, F-PTK6 or MF-PTK6. Soft agar colony growth was assessed after four weeks. A representative experiment of three replicate experiments using triplicate wells for each cell line is shown. Error bars indicate standard deviation. Western blot analysis shows levels of expression of PTK6 in each cell line.

To complement the loss-of-function studies, we examined the effect of overexpressing PTK6 in both MCF-10A cells and immortalized human mammary epithelial cells (HMLE). Overexpression of PTK6 in parental MCF-10A cells using either a FLAG-tagged wild type PTK6 (F-PTK6) or myristylated, membrane targeted PTK6 (MF-PTK6), was sufficient for a modest, but significant, suppression of cell death upon detachment from matrix (Figure 3A). MCF-10A cells overexpressing MF-PTK6 consistently displayed a greater degree of anoikis resistance when compared to cells overexpressing F-PTK6. PTK6 kinase activity is important for suppression of anoikis of MCF-10A cells, as overexpression of a kinase-inactive MF-PTK6 (KD-MFPTK6) did not significantly rescue these cells from matrix detachment-induced cell death (Figure 3B).

Overexpression of PTK6 alone (MF-PTK6 or F-PTK6) in HMLE was not sufficient to significantly promote colony formation in soft agar, another measure of anchorage-independent survival (Figure S2). However, PTK6 was able to enhance soft agar colony growth induced by other oncogenes that activate the PI3-K or Erk pathways (Figure 3C). Utilizing a cell system similar to that described previously in which activated Akt and MEK are used to substitute for Ras activation in transformation of immortalized HMEC [22], we observed that overexpression of PTK6 in immortalized HMLE expressing either activated MEK (HMLE-M) or myristylated Akt (HMLE-A) significantly enhanced colony formation (Figure 3B). Interestingly, membrane-targeted PTK6 was as effective, if not more effective, than wild type PTK6 in enhancing colony formation. Although PTK6 lacks a native myristylation sequence (in contrast to other Src family kinases), PTK6 has been reported to localize to membrane ruffles and associate with membrane-bound receptors such as EGFR and ErbB2 [23]–[25]. Membrane-localization may therefore play a critical role in the collaborative transforming functions of PTK6.

### PTK6 modulates anoikis sensitivity of breast and ovarian cancer cells expressing IGF-1R

**Figure 4:**
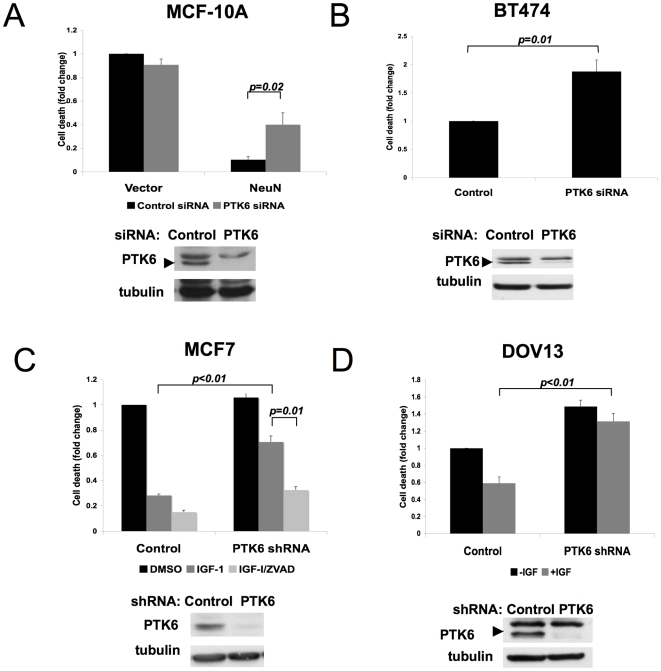
PTK6 downregulation inhibits anchorage-independent survival of breast and ovarian cancer cells. A) PTK6 downregulation inhibits survival of Her2^+^ cells. The effect of PTK6 downregulation on anchorage-independent survival of MCF-10A cells overexpressing ErbB2 was assessed. Cell death was assessed as in Figure 1. The average fold change in cell death from three independent experiments is shown. Error bars indicate standard deviation. B) PTK6 downregulation inhibits survival of Her2+ breast cancer cells. The effect of downregulating PTK6 on anchorage-independent survival of BT474 cells was assessed. Cell death was assessed as in Figure 1. The average fold change in cell death from three independent experiments is shown. Error bars indicatestandard deviation. C) Downregulation of PTK6 inhibits IGF-1-induced survival of MCF-7 breast cancer cells. The effect of PTK6 downregulation on anchorage-independent survival of IGF-1 (100ng/ml) stimulated MCF-7 breast cancer cell line was assessed in the presence or absence of ZVAD-fmk (25µM). Cell death was assessed as in Figure 1. The average fold change in cell death from three independent experiments is shown. Error bars indicate standard deviation. D) Downregulation of PTK6 inhibits IGF-1 induced survival of DOV-13 ovarian cancer cells. The effect of PTK6 downregulation on anchorage-independent survival of DOV-13 ovarian cancer cells was determined in the presence or absence of IGF-1 (100ng/ml). Cell death was assessed as in Figure 1. The average fold change in cell death from three independent experiments is shown. Error bars indicate standard deviation.

We sought to determine whether PTK6 plays a critical role in modulating anchorage-independent survival of breast and ovarian cancer cells in which PTK6 is highly expressed, particularly those which co-express IGF-1R. Examination of a panel of cell lines revealed that PTK6 was abundantly expressed in several Her2^+^ breast cancer lines, as previously reported (Figure S3A). Downregulation of PTK6 was associated with increased anoikis of BT474 cells and ErbB2 overexpressing MCF-10A cells (NeuN), lines in which anchorage-independent survival is induced via the ErbB2 growth factor receptor, supporting a role for PTK6 in survival of Her2^+^ cells (Figures 4A and 4B). PTK6 was also highly expressed in several Her2^−^, ER^+^ cell lines, including MCF-7 (Figure S3A). MCF-7 cells express high levels of endogenous IGF-1R, thereby providing another context for evaluating the role of PTK6 in IGF-1 induced, anchorage-independent survival. Stimulation of MCF-7 cells with IGF-1 rescued cells from anoikis, which was significantly reversed by downregulation of PTK6 (Figure 4C). This death was inhibited by the caspase inhibitor ZVAD-fmk, again supporting a role for PTK6 in modulating apoptosis of breast cancer cells under matrix-detached conditions. These results support an expanded role for PTK6 in growth factor receptor-induced anoikis resistance of breast cancer cells.

Downregulation of PTK6 also prevented IGF-1 stimulated anoikis resistance of DOV-13 ovarian cancer cells, which co-express PTK6 and IGF-1R (Figure 4D). There is increasing evidence supporting a role for IGF-1 signaling in ovarian tumor progression [26], [27]. Furthermore, PTK6 has previously been reported to be amplified and overexpressed in a significant number of ovarian cancers although its functions in ovarian tumorigenesis have not been studied [18]. In contrast to normal human ovarian surface epithelial (HOSE) cells which do not express detectable levels of PTK6, DOV-13 cells express higher levels of PTK6 protein ([18] and Figure S3B). While DOV-13 cells are relatively sensitive to anoikis when compared to HOSE cells, IGF-1 stimulation enhances survival in suspension (Figure 4D). Downregulation of PTK6 reverses this IGF-1 induced anchorage-independent survival. Interestingly downregulation of PTK6 also enhanced basal levels of death, suggesting that it may also play a critical role in anchorage-independent survival of DOV-13 cells distinct from its role in IGF-1-stimulated anoikis resistance.

### Regulation of IGF-1R signaling by PTK6

**Figure 5:**
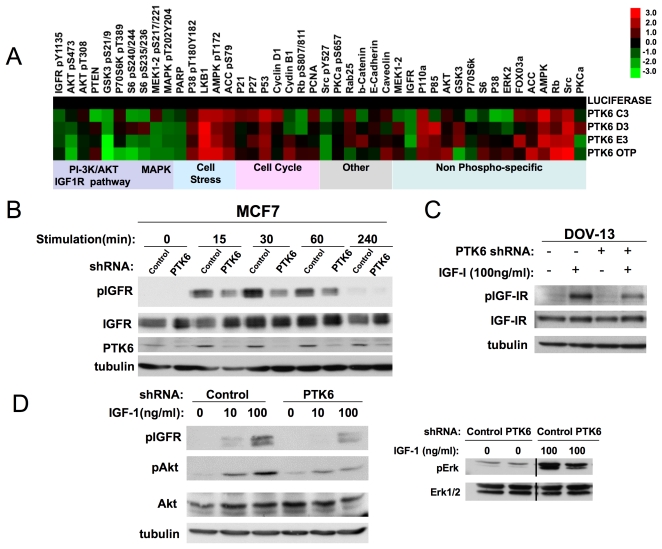
PTK6 downregulation affects IGF-1 dependent signaling pathways. A) Reverse-phase protein arrays (RPPA) reveal changes in IGF-1-stimulated signaling with PTK6 downregulation. RPPA analysis was performed on lysates of IGF-1R cells overexpressing Bcl2 (IGF-1R/Bcl2) that were transfected with control or PTK6 siRNA oligonucleotides in suspension cultures containing IGF-1 (100ng/ml). The heatmap represents values normalized to luciferase control from transfections performed in triplicate. B) shRNA-mediated downregulation of PTK6 in MCF-7 cells decreases ligand-stimulated IGF-1R phosphorylation. MCF-7 cells expressing control or PTK6 shRNA were stimulated in suspension cultures for the indicated times with 100ng/ml IGF-1. Lysates were probed with the indicated antisera. C) DOV-13 cells cultured on adherent plates were stimulated with 100ng/ml IGF-1 for 20 minutes. Lysates were probed with the indicated antisera. D) PTK6 downregulation inhibits IGF-1- stimulated activation of Akt and Erk/MAPK. Lysates of MCF-7 cells stimulated with the indicated concentrations of IGF-1 for 60 minutes (left panel) or 15 minutes (right panel) in suspension cultures were probed with the indicated antisera.

**Figure 6:**
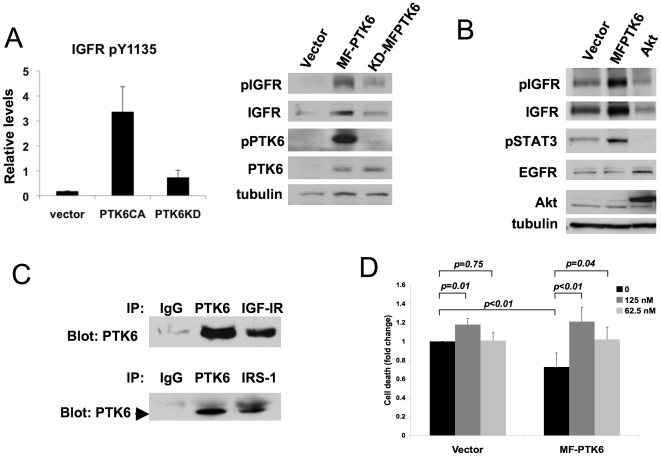
Overexpression of PTK6 enhances anchorage-independent survival by regulating IGF-1R phosphorylation. A) PTK6 overexpression increases phosphorylation of IGF-1R. (Left) MCF-10A cells overexpressing a constitutively active (CA) or kinase-inactive (KD) PTK6 were grown in suspension cultures for 48 hours in the presence of IGF-1 (100ng/ml). Lysates were prepared and probed using RPPA. The values represent the average normalized expression values derived from triplicate samples. Error bars indicate standard deviation. (Right) Lysates prepared from suspension cultures of MCF-10A cells overexpressing vector control, MF-PTK6 or kinase-inactive MF-PTK6 (KD-MFPTK6) were probed with the indicated antisera. B) Lysates prepared from suspension cultures of MCF-10A cells overexpressing vector control, MF-PTK6 or wild type Akt in the presence of IGF-1 (100ng/ml) were probed with the indicated antisera. C) PTK6 co-precipitates with IGF-1R and IRS-1 adapter protein. Lysates of MCF-10A cells overexpressing IGF-1R and wild type PTK6 were immunoprecipitated with the indicated antisera and immunoblotted with anti-PTK6 antibody. D) Anchorage-independent survival stimulated by PTK6 overexpression is suppressed by IGF-1R kinase inhibition. MCF-10A cells overexpressing vector control or MF-PTK6 were grown in suspension cultures containing IGF-1 (100ng/ml) for 48 hours in the absence or presence of BMS-536924 at the indicated concentrations. Cell death was assessed as described in Figure 1. The fold change in cell death represents the average of three independent experiments. Error bars indicate standard deviation.

To begin to elucidate the mechanisms by which PTK6 modulates anchorage-independent survival, we utilized reverse-phase protein arrays (RPPA) to identify signaling molecules that are altered by downregulation or overexpression of PTK6 (Figures 5A, 6A). IGF-1R cells overexpressing Bcl2 (IGF/Bcl2) were transfected with multiple different siRNA oligonucleotides targeting PTK6 in suspension cultures in order to minimize changes in phosphoprotein signatures resulting secondarily from apoptosis. Decreased expression of PTK6 was associated with changes in several proteins that regulate cell metabolism and growth factor signaling pathways. Downregulation of PTK6 induced an increase in metabolic “stress” as indicated by the enhanced signal intensities for AMPK and phospho-AMPK, and its substrate phospho-ACC. In contrast, there was a decrease in the signal intensities for phospho-Akt, phospho-Erk and phospho-S6, suggesting a widepspread impact of PTK6 downregulation on signaling pathways, either as a primary or secondary consequence. Interestingly, we observed that the signal intensities for phospho-IGF-1R and total IGF-1R were also decreased on the protein array, which raised the possibility that PTK6 modulates activation and/or expression of the receptor itself, thereby influencing multiple downstream signaling molecules. Western analyses of lysates derived from IGF-1 stimulated MCF-7 cells confirmed that downregulation of PTK6 resulted in decreased levels of IGF-1 receptor phosphorylation of known autophosphorylation sites (tyrosine 1131, 1135 and 1136) which are required for maximal IGF-1R kinase activity (Figure 5B). This decrease in IGF-1R phosphorylation was not associated with a decrease in IGF-1R levels. The effects of PTK6 downregulation on IGF-1 receptor phosphorylation are unlikely to be secondary to changes in viability induced by matrix deprivation as these changes were observed in IGF-1R cells co-expressing Bcl2 which are protected from anoikis induced by PTK6 downregulation. Furthermore, similar changes in IGF-1 receptor phosphorylation were observed with downregulation of PTK6 in attached DOV-13 ovarian cancer cells (Figure 5C). PTK6 downregulation-induced decreases in signaling downstream of IGF-1R (e.g. Akt, Erk) were also confirmed in MCF-7 cells acutely stimulated with IGF-1 in suspension cultures (Figure 5D).

The effect of overexpressing PTK6 on IGF-1R phosphorylation was also examined (Figure 6). In the RPPA studies an enhanced signal for phospho-IGF-1R (phosphorylated at tyrosine 1131/1135/1136) was detected with overexpression of active, PTK6 (Figure 6A). By western analyses, an increase in IGF-1R phosphorylation was observed in MCF-10A cells overexpressing a catalytically active, membrane-targeted PTK6 (MF-PTK6) (Figure 6A). Increased levels of total IGF-1R accompanied the enhanced phosphorylation, whereas downregulation of PTK6 affected phosphorylation independent of receptor levels. Overexpression of kinase-inactive PTK6 (KD-MFPTK6) also slightly enhanced IGFR phosphorylation, although not to the level observed with MF-PTK6. No change in total receptor level was observed with overexpression of KD-MFPTK6. PTK6 overexpression does not have a general effect on membrane-associated receptor tyrosine kinases in suspension, as levels of EGFR, previously reported to activate PTK6, were unchanged (Figure 6B). Moreover, the enhanced IGF-1R phosphorylation is unlikely to be simply secondary to enhanced viability of MCF-10A cells in suspension cultures as overexpression of Akt, which potently inhibits anoikis, did not result in similar changes in IGF-1R levels or phosphorylation (Figure 6B).

Previous studies have shown that PTK6 is able to form complexes with EGF receptor family members and PTK6 couples these receptors to downstream signaling pathways [28]. PTK6 could also regulate IGF-1R signaling by physical association with the receptor complex, as PTK6 was previously shown to associate with IRS-4 in HEK-293 cells [29]. In MCF-10A cells overexpressing IGF-1R and PTK6, PTK6 co-precipitated with IGF-1R (Figure 6C). PTK6 was also detected in immune complexes containing IRS-1, suggesting that PTK6 may regulate IGF-1R by associating with the receptor∶adapter complex.

To determine whether IGF-1R kinase activity is critical for the suppression of anoikis induced by PTK6 overexpression, we assessed the effect of BMS-536924, an IGF-1R/insulin receptor inhibitor, on the anchorage-independent survival of MCF-10A cells overexpressing MF-PTK6. Cells were cultured in suspension in the presence of sub-saturating doses of the inhibitor, which reversed the enhanced survival induced by PTK6 (Figure 6D and Figure S4). Collectively, these results provide evidence for PTK6's role in anchorage-independent survival via regulation of IGF-1R and IGF-1 stimulated signaling.

### PTK6 is amplified and expressed in specific subtypes of human breast tumors

**Figure 7:**
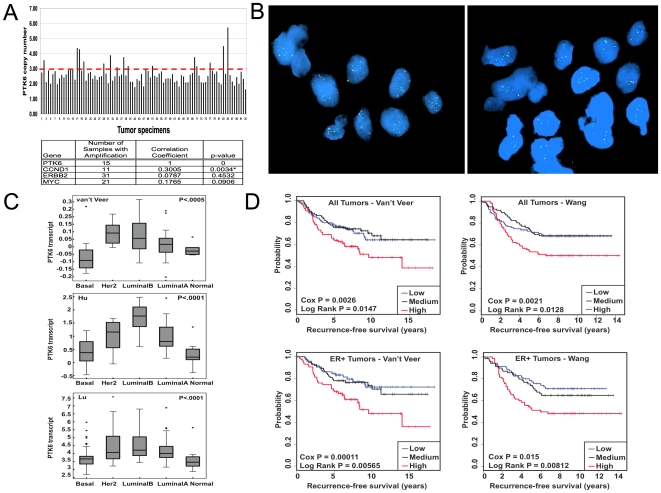
PTK6 copy number gain and overexpression in primary human breast tumor samples. A) (Top) SNP array analyses of 93 breast tumors demonstrates PTK6 copy number gain in a subet of tumors.The x-axis represents individual breast tumor specimens; the y-axis represents copy number. The red line indicates copy number = 3 (Bottom) Correlation analysis with other amplified genes is shown. The table lists the number of tumors with PTK6 copy number gain (copy number≥3.0), the Pearson product-moment correlation for each pair of variables and the associated P-values. B) FISH analysis demonstrates PTK6 copy number gain. Two human breast tumors were selected for FISH analyses based on high PTK6 transcript expression levels in microarray analyses. FISH analysis was performed using a probe specific for PTK6 (green) or a control chromosome 20 centromeric probe (pink). C) PTK6 mRNA is differentially expressed in human breast cancer subtypes. Publicly available datasets were used to compare levels of PTK6 mRNA in breast cancer subtypes (basal, HER2/ERBB2^+^, Luminal A, Luminal B) and normal samples. P-values were calculated using ANOVA in JMP 7.0 software. D) PTK6 mRNA level is a predictor of prognosis in human breast cancer. Kaplan-Meier curves depict the probability of breast cancer recurrence based on relative levels of PTK6 transcript expression. Probabilities for the entire cohort (top) as well as the ER^+^ subgroup (bottom) were determined. The Cox regression test evaluates the association of PTK6 level with patient outcome treating the level of PTK6 as a continuous variable. The log-rank test evaluates whether there are significant differences between any of the three groups. Data sets from Van't Veer et al. (Van't Veer et al., 2002) and Wang et al.(Wang et al., 2005) were analyzed.

PTK6 may promote human cancer growth by modulating signaling via growth factors receptors, such as IGF-1R and ErbB2. PTK6 has been reported to be expressed in multiple tumor types, including breast and ovarian cancer [14]–[16], [18]. This expression pattern may be in part due to copy number gain as PTK6 maps to Chromosome 20q13.3, a region frequently amplified in breast tumors. Recently, Xiang et al. reported coamplification of PTK6 in Her2^+^ breast tumors [23]. We examined PTK6 copy number in a cohort of 93 breast tumors. Genomic DNA extracted from a series of breast tumors was hybridized to SNP (Single Nucleotide Polymorphism) arrays [30] (Figure 7A). PTK6 copy number gain (copy number greater than or equal to 3.0) was observed in 15 of 93 breast tumors (15%) in SNP analysis. Most PTK6 copy number gains are modest and with the exception of one tumor, are broad (∼2.8Mbp) gains (Figure S5). Fluorescence in situ hybridization of tumors selected on the basis of elevated PTK6 signal intensity in microarray analyses showed an increase in the number of hybridized signals with the PTK6-specific probe (Figure 7B). For some tumor cells within a sample, there was also an observed increase in the number of hybridized signals with the Chromosome 20 centromere-specific probe, suggesting that in some cases apparent PTK6 copy number gains may be due to increased numbers of chromosome 20. A statistically significant correlation between PTK6 copy number gain and cyclin D1 amplification was also observed (Figure 7A). However, in contrast to a previous report [23], a correlation with ErbB2 amplification was not observed in the set of tumors analyzed for this study.

We examined the relative expression of PTK6 mRNA in different breast tumor subtypes (Figure 7C). In previously published microarray analyses of breast tumor specimens downloaded from Oncomine, PTK6 transcript levels were elevated in nearly all breast tumor subtypes with the exception of basal-like tumors. PTK6 expression was the highest in both Her2^+^ and high-grade ER^+^ (Luminal B) tumors. Many Luminal B tumors also express high levels of IGF-1R transcript (data not shown).

Elevated levels of PTK6 mRNA expression are associated with prognostic significance. In two large breast cancer patient cohorts with long-term follow up, tumor specimens were stratified based on PTK6 expression levels. Patients with tumors with the highest expression levels of PTK6 mRNA transcript in each cohort were less likely to be recurrence-free over long-term follow up (Figure 7D, top). This prognostic significance was observed independent of estrogen receptor or nodal status in multivariate analyses (data not shown). High PTK6 transcript levels were also associated with adverse outcomes for the subset of patients with ER^+^ tumors in both cohorts (Figure 7D, bottom). This prognostic significance was observed in multiple other cohorts that utilized different array platforms (Figure S6).

## Discussion

In contrast to normal epithelial cells, most cancer cells have acquired the ability to grow in an anchorage-independent manner. In this report, we identified PTK6 as a gene that is critically involved in IGF-1R-stimulated anchorage-independent survival using a highly validated siRNA screen. PTK6 was also found to be required for anchorage independence of breast and ovarian cancer cells. These findings were supported by gain-of-function studies showing that PTK6 is an effective “collaborative oncogene”, efficiently enhancing transformation in combination with other activated oncogenes. Insights into the mechanisms whereby PTK6 induces anoikis resistance were provided by high throughput protein arrays and subsequent analyses, which demonstrated that loss of PTK6 had broad effects on the major signaling pathways activated by IGF-1R as well as IGF-1R itself. Subsequent studies confirmed that PTK6 regulates the expression and the activity of IGF-1R. Effects of PTK6 on anchorage-independence could contribute to the poor outcomes associated with high PTK6 transcript levels that we found in multiple cohorts of breast cancer patients.

Our studies indicate that PTK6 regulates anchorage-independent survival through suppression of caspase-mediated apoptosis, as downregulation of PTK6 increased PARP cleavage and resulted in cell death that was inhibited by ZVAD-fmk. Interestingly, Harvey et al. recently reported that downregulation of PTK6 in the matrix-deprived T47-D breast cancer cells induces autophagy, but not apoptosis [31]; however, they did not assess whether autophagy was responsible for cell death. Matrix detachment has been shown to cause metabolic impairment [32] and induce autophagy associated with activation of AMPK, an enzyme that senses metabolic stress [33]. Interestingly, inhibition of autophagy enhances apoptotic cell death in MCF-10A cells [33], suggesting that autophagy promotes epithelial cell survival, rather than cell death, in matrix-detached MCF-10A cells.

RPPA analyses in this study demonstrated that AMPK and its target protein ACC were activated by PTK6 downregulation, indicating that loss of PTK6 also causes metabolic stress. In addition, although cell death induced by PTK6 downregulation was inhibited by ZVAD-fmk, the metabolic impairment was not (data not shown). While autophagy can be an adaptive survival mechanism for cells under stress it can also progress to cell death (reviewed in [34], [35]). It is possible that both autophagy and apoptosis play roles in cell death resulting from PTK6 downregulation and the relative contribution may differ depending on cellular context, levels of anti-apoptotic proteins and duration of matrix deprivation. Our studies together with previous reports [36], [37] indicating that PTK6 may regulate multiple signaling molecules infer that there may be multiple mechanisms by which PTK6 regulates cell growth and survival.

Overexpression of PTK6 enhanced anchorage-independent survival. Although PTK6 is distantly related to the Src family of tyrosine kinases with an SH3, SH2 and catalytic domain, it lacks a native myristylation signal shared by most members of this class [14], [38]. The ability of myristylated PTK6 to effectively enhance anchorage-independent survival points to a potentially critical role for membrane localization in this oncogenic function of PTK6. During the preparation of this manuscript, Kim and Lee also reported that myristylated PTK6, but not nuclear localized PTK6, promoted proliferation, migration, and colony formation of HEK293 cells [39]. Membrane localization is not a general requirement for all src-family kinases, as a related kinase Frk, which also lacks a native myristylation sequence, failed to enhance soft agar colony growth when targeted to the membrane (unpublished data). Thus subcellular localization likely determines access to specific substrates and interacting partners that modulate PTK6's role in tumorigenesis.

While the ability of PTK6 to enhance signaling downstream of EGF receptor family kinases is well established [24], [28], our studies are the first to highlight the ability of PTK6 to regulate other growth factor receptor kinases, namely IGF-1R. Complementary protein arrays performed on cells with either overexpression or downregulation of PTK6 revealed an effect of PTK6 on IGF-1 receptor phosphorylation and function. This ability of PTK6 could underpin its critical role in a subset of breast tumors which do not express substantial levels of ErbB receptor family kinases, but rather express high levels of both IGF-1R and PTK6. Specifically, we observed a trend for high- grade ER^+^ (i.e. Luminal B) tumors to express high levels of both of these transcripts (data not shown); therefore, PTK6 may contribute to the growth and survival of these tumors by modulating IGF-1R signaling.

Whether the effects of PTK6 on IGF-1R function involve direct interactions with the receptor remain to be addressed. The ability of PTK6 to regulate the IGF-1 receptor does not appear to be dependent on a secreted factor as conditioned media from cells in which PTK6 expression was modulated failed to induce any change in IGF-1R (data not shown). The ability of PTK6 to co-precipitate with both the IGF-1 receptor and IRS-1 suggests that PTK6 is able to associate with the receptor complex. Interestingly, v-Src has been reported to directly phosphorylate the beta chain of the IGF-1 receptor at the corresponding autophosphorylation sites in rat fibroblasts with subsequent activation of IGF-1R kinase, and IGF-1R is required for v-Src transforming activity [40]. PTK6, a Src family member, may similarly phosphorylate IGF-1R directly following recruitment to the membrane.

Although both gain- and loss-of-function approaches used in this report highlighted a positive role for PTK6 in anchorage-independent survival, other studies utilizing mouse intestinal cells and rat fibroblasts suggest that PTK6 may be required for cell death triggered by specific stimuli such as DNA damage [41], [42]. There may be tissue-specific functions of PTK6 depending on differential substrate/binding partner availability. In addition, given the critical role that subcellular localization may play in oncogenic vs. non-oncogenic activity, the ability of PTK6 to be recruited to the membrane by any mechanism may determine its ultimate role in cell survival.

PTK6 is expressed in multiple human tumor types, including breast and ovarian tumors [14]–[16], [18]. PTK6 maps to a chromosomal region (20q13.3) that is frequently amplified in breast tumors and amplicons spanning this region variably contain other suspected oncogenes, such as TDE1, NCoA3, BCAS4, and ZNF217. The PTK6 copy number gain detected in our study is relatively modest. However, it is possible that low levels are sufficient to contribute to tumorigenic phenotypes collaboratively, as we have shown that PTK6 effectively collaborates with other oncogenic signaling pathways. In multiple microarray studies, elevated PTK6 mRNA expression was most highly associated with specific breast cancer subtypes (Her2^+^ and Luminal B/high grade ER^+^). Although we have shown that PTK6 expression is critical for anchorage-independent survival of representative cell lines of these subtypes, the full range of functions attributable to PTK6 expression in these specific subtypes (e.g. regulation of sensitivity to chemotherapy or endocrine therapy, proliferation) remains to be addressed.

In our study we analyzed the prognostic significance of elevated PTK6 transcript expression in multiple large cohorts of patients with breast cancer. Our finding that elevated expression of PTK6 transcript correlates with adverse outcomes for patients with breast cancer in all cohorts analyzed is consistent with a recently reported correlation between PTK6 mRNA levels and tumor grade based on analyses of 44 tumors [31]. However, this association of PTK6 with adverse outcome contrasts with findings of another group who reported (1) no correlation between PTK6 mRNA expression levels and disease-free survival, and (2) a positive correlation between PTK6 protein expression (determined by immunohistochemistry) and long-term disease-free survival. [43]–[45]. The exact basis for this discrepancy with our findings for PTK6 mRNA levels is not clear, although the analyses of Aubele et al. were all performed on a single cohort. Their finding with respect to PTK6 protein expression is intriguing; the lack of correlation between immunohistochemical signal intensity and either PTK6 FISH signals or transcript levels suggests that PTK6 protein levels may be regulated post-transcriptionally. Further complicating the studies of prognostic significance is the finding that subcellular localization of PTK6 protein may critically regulate oncogenic activity; in studies of prostate tumor cells, cytoplasmic localization of PTK6, in contrast to nuclear expression, was associated with poor differentiation and androgen independence, which are adverse prognostic factors [46]. Thus, immunohistochemical analyses of breast tumor specimens with attention to PTK6 expression in specific subcellular compartments could be informative.

PTK6 is an attractive potential therapeutic target for breast and ovarian cancer for several reasons. The rather restricted expression of PTK6 in normal tissues and the prevalence of PTK6 copy number gain and high expression in some breast and ovarian tumors improve the chance of selectively targeting malignant cells. PTK6 overexpression could contribute to resistance to currently available therapies, such as those targeting Her2 amplified tumors [23]. Inhibition or downregulation of PTK6 may also prove to be an effective strategy for enhancing the response of high-grade ER^+^/Luminal B breast cancers to endocrine therapy or overcoming acquired resistance to these agents. Validation of PTK6 as a viable target will depend on identifying those tumors in which PTK6 is functionally active and determining the specific substrates and domains of PTK6 that are required for its oncogenic activity.

## Materials and Methods

### Reagents, cells and cell culture

MCF-10A, ZR751, MCF-7, BT474, DOV-13, Hey C2 and human immortalized mammary epithelial cells (HMEC) were obtained from ATCC (Manassas, Virginia). MCF-10A cells overexpressing IGF-1R receptor (IGF-1R) have been previously described [21]. MCF-10A cells and IGF-1R cells were cultured, as previously described [21], [47]. ZR-751 cells were cultured in RPMI 1640 supplemented with 10% fetal bovine serum. MCF-7 cells were cultured in DMEM supplemented with 10% FBS. Ovarian cancer lines were cultured in RPMI-0 supplemented with 10% fetal bovine serum and penicillin/streptomycin. Three-dimensional (3D) Matrigel™ cultures were performed as described previously [21]. ZVAD-fmk, a pan-caspase inhibitor, was purchased from BD Biosciences. IGF-1 receptor kinase inhibitor (BMS-536924) was purchased from Selleck and solubilized in DMSO. Antiserum or monoclonal antibodies directed against the following proteins were obtained from the indicated suppliers: Cell Signaling Technologies: phospho-IGF-1R (tyrosine 1131/1135/1136), phospho-Akt (Ser 473), Akt, cleaved PARP, phospho-STAT3, EGFR and Akt1; Santa Cruz Biotechnology: IGF-1R, PTK6, Erk2, tubulin; Biosource: phospho-Erk1/2; Millipore: IRS-1 and phospho-Brk; and Chemicon: Laminin 5,γ2 subunit (LAMC2). Alamar Blue was purchased from Biosource.

### Constructs, virus production and stable cell line generation

The constructs for PTK6 (kinase-active and kinase-inactive) were generated by cloning into pMSCV-puro. The construct encoding short hairpin RNA sequences targeting PTK6 was obtained from Open Biosystems (Catalog # TRCN0000021552). pBP-MF-PTK6 and pBP-F-PTK6 were obtained from the Harvard Institute of Proteomics (gift of Haley Hieronymus and Jesse Boehm). Viral packaging 293T or GPG-293T cells were transfected according to standard protocols. Viral supernatant was collected 36 and 60 hrs post-transfection. Immortalized HMECs or MCF-10A cells were infected in the presence of 2µg/ml polybrene with the viral supernatant or were spin-infected for 30 minutes at 2250 rpm. After three hours, media was changed on the target cells, which were then allowed to recover overnight, followed by another round of infection. Forty-eight hours post-infection, the target cells were exposed to puromycin (0.5–1 µg/ml for 48 hrs) to select for infected cells.

### Anchorage-independent viability screen

A library consisting of two individual siRNA sequences targeting human kinases were obtained from Qiagen (v.1.0). MCF-10A cells overexpressing IGF-1R were trypsinized and plated in 96 well plates pre-coated with polyhema (Sigma). Cells were transfected using Oligofectamine in quadruplicate wells with individual siRNA duplexes from the Qiagen kinase siRNA library. Cells were cultured for an additional 72 hours in suspension and assayed for Alamar reducing potential on a multiplate reader according to the manufacturer's protocol.

In the counter screen, parental MCF-10A cells cultured on polyhema plates were transfected with siRNA duplexes. After four hours the media was supplemented with Matrigel™ to a final concentration of 2%. After an additional 72 hour incubation, Alamar blue reduction was assayed as above. The ratio between the Alamar blue score in IGF1R-overexpressing cells to the score for MCF10A cells grown in Matrigel was calculated. This ratio was used to select candidates for testing additional siRNA reagents.

### Assessment of cell death

Cell death in suspension cultures was assessed using the Cell Death ELISA kit (Roche Diagnostics).according to the manufacturer's instructions.

### Soft agar colony assays

The bottom layer of soft agar contained 0.6% agar with DMEM and 5% serum. Fifteen thousand cells were seeded per well on the top layer that contained 0.3% agar in MEGM. Cells were seeded in triplicate wells of six-well plates. Wells were re-fed every two weeks and colonies were counted at four weeks post-plating. At each time point, an image of each well was taken at 6× magnification and processed using the software Image J. Colonies larger than 50 sq. pixels were counted.

### SNP array analysis

Breast tumors for SNP array analysis were collected from de-identified cases using protocol #98-229 approved by the Institutional Review Board of the Dana-Farber Cancer Institute. SNP array analyses were performed by the Dana-Farber Microarray Core and by the Broad Institute using Affymetrix 250K StyI arrays, according to methods described by Nikolsky et al [30]. The raw .CEL files were normalized and copy number of each SNP was determined using the GenePattern software [30]. Raw SNP copy numbers were smoothed by a segmentation algorithm using the DNA copy package (available at www.bioconductor.org). Copy numbers of each gene were estimated by averaging copy numbers from all SNPs found within the gene structure and flanking 100-kbp regions. All of the raw data were deposited into Oncomine and Gene Expression Omnibus (GSE19399) and are publicly available: (https://www.oncomine.com/resource/login.html and http://www.ncbi.nlm.nih.gov/sites/entrez?db=gds&term=GSE19399Accession&cmd=search).

### FISH analysis of tumors

A fosmid probe specific for PTK6 and a Chromosome 20 centromere probe were obtained from CHORI/BacPac library. Hybridization and analyses were performed by the Dana-Farber/Harvard Cancer Center Cytogenetics Core Facility (P30 CA006516).

### Microarray analyses

#### Breast tumor subtype analyses

Boxplots showing the level of PTK6 mRNA in breast tumor subtypes were derived from three data sets: (1) GSE1992 downloaded from the Gene Expression Omnibus (GEO, http://www.ncbi.nlm.nih.gov/projects/geo/) [48], (2) Rosetta Inpharmatics (http://www.rii.com/publications/2002/vantveer.html) [49] and (3) a gene expression array (Affymetrix U133) of 129 sporadic primary invasive breast tumors which includes tumors published previously [50], [51]. Boxplots were generated in Matlab and p-values (ANOVA) were calculated in JMP 7.0. Classification of tumors in the Van't Veer data set was obtained from GEO (GSE4382) [52].

For GSE5460, raw expression values obtained from Affymetrix GENECHIP software were additionally analyzed using DNA-Chip analyzer (dChip) custom software (www.dchip.org). Parallel data of 12 normal breast organoids RNA samples and 7 bulk normal breast tissue specimens were used as normal control. Array probe data were normalized to the mean probe expression level across the cohort; thus, expression levels are relative to a normalized average. Gene filtering and hierarchical cluster analysis were performed using dChip software to identify subtypes of breast tumors using the intrinsic gene list defined by Sorlie et al. [52]. Two PTK6 probes in the array 206482_at and 1553114_a_at were used for analysis of PTK6 transcripts level. The relative levels of hybridization for the two probes were highly correlated (linear regression R = 0.83).

#### Kaplan-Meier curves

For the Van't Veer data set, normalized PTK6 expression values and time to recurrence data were downloaded from the Rosetta website (http://www.rii.com/publications/2002/vantveer.html). Samples were divided into three equal tertiles: 98 tumors with highest PTK6 level (>0.053); 99 tumors with intermediate PTK6 level (between −0.021 and 0.056) and 98 tumors with lowest PTK6 level (<−0.021). For the ER^+^ plots, only the 226 samples classified as ER+ by Van't Veer et al. were used.

For the Wang set, PTK6 expression values and time to recurrence data were downloaded from the Gene Expression Omnibus (GEO, GSE2034). Samples were divided into three equal tertiles: 95 tumors with highest PTK6 level (>242.3); 96 tumors with intermediate PTK6 level (between 126.3 and 243.7) and 95 tumors with lowest PTK6 level (<126.9). For the ER^+^ plots, only the 209 samples classified as ER+ by Wang et al. were used.

For all survival curves, the y-axis (probability) is defined as the frequency of relapse-free survival. Kaplan-Meier analyses were carried out using survival package within R language. P-values were derived using the log-rank test and by fitting a Cox proportional hazards regression model. Multivariate Cox proportional hazards analysis was performed for PTK6 and clinical prognostic factors including estrogen receptor (ER),HER2 oncogene, axillary lymph node involvement.

### Immunoprecipitations, and Western analyses

Cells were lysed in RIPA lysis buffer (1%Triton X-100, 1%NaDOC, 0.1%SDS, 20mM Tris pH 7.5, 150 mM NaCl, 1mM EDTA) or RPPA lysis buffer (1% Triton X-100, 50mM HEPES, 150mM NaCl, 1.5mM MgCl2, 1mM EDTA, 100mM NaF, 10mM NaPP, 10% glycerol, 1mM PMSF, 1mM Na_3_V0_4_) supplemented with protease and phosphatase inhibitors (1µg/ml leupeptin, 1 µg/ml aprotinin, 1 µg/ml pepstatin, 10 µg/ml PMSF, 1 mM NaVO_4_, 1mM NaF) for 30 minutes at 4°C. Lysates were clarified by centrifugation and supernatants were collected. Proteins were resolved by 8–10% SDS-PAGE gel electrophoresis and immunoblotted using standard techniques.

### Reverse phase protein array (RPPA) analysis

Serially diluted cell lysates were printed on nitrocellulose-coated slides and probed with specific antibodies as previously reported [53]. Signal intensity data were collected and analyzed using software specifically developed for RPPA analyses (http://www.vigenetech.com). The normalized linear values for each sample were divided by the trimmed mean (25%) of the values for that sample. Each value was standardized using a modified z-score.
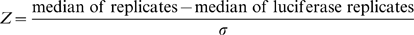
Heatmaps were generated using Cluster 3.0 and Java TreeView 1.1.1.

### Phase contrast microscopy

Cells grown in monolayer cultures, as well as 3D acinar structures were visualized at 20°C using a Nikon TE300 microscope equipped with a CCD camera, using a 4×/0.13 objective. Images were acquired using ImageJ software, converted to TIFF images and arranged using Adobe Photoshop 7.0.

### Immunofluorescence analyses and confocal microscopy

Acinar structures were fixed in 2% formalin (Sigma) at room temperature for 20 minutes and permeabilized in 0.5% Triton X-100 in PBS for 10 min at 4°C. Immunostaining of acinar structures was carried out as previously described [47] and imaged at 20°C. DAPI was purchased from Molecular Probes. Confocal analyses were performed using the Nikon TE2000 microscope with the C1plus confocal microscope system equipped with krypton-argon (488 line) and HeNe (543 and 633 lines) lasers. Structures were analyzed with a 40×/1.3 objective and images were acquired using the Nikon C1 Confocal software. All images were converted to TIFF format and arranged using Adobe Photoshop 7.0.

## Supporting Information

Figure S1 siRNAs targeting PTK6 identified by the screen inhibit anchorage-independent survival of IGF-1R cells. A) Parental and IGF-1R overexpressing MCF-10A cells transfected with control or PTK6 siRNA (OTP, Dharmacon) were cultured in suspension for 48 hours. Lysates were probed with the indicated anti-sera. B) Western analysis demonstrates down regulation of PTK6 expression in IGF-1R cells by siRNAs targeting PTK6 identified by the screen to induce changes in viability. IGF-1R cells transfected with multiple PTK6 siRNAs, including a pool of siRNAs from a distinct vendor (OTP, Dharmacon), were cultured in suspension for 48 hours. Lysates were prepared and probed with the indicated anti-sera. C) siRNAs targeting PTK6 inhibit anchorage-independent survival of IGF-1R cells. IGF-1R cells transfected with multiple PTK6 siRNAs, including a pool of siRNAs from a distinct vendor (OTP, Dharmacon), were cultured in suspension for 48 hours. Cell death was assessed as described in Figure 1. The average fold change from three independent experiments is shown. Error bars indicate standard deviation.
(0.24 MB TIF)

Figure S2 Overexpression of PTK6 alone in HMEC does not enhance growth in soft agar. Immortalized human mammary epithelial cells (HMLE) overexpressing vector control, F-PTK6 or MF-PTK6, were seeded in soft-agar, and colony formation was assessed after four weeks. A representative experiment of three replicate experiments using triplicate wells for each cell line is shown.
(0.08 MB TIF)

Figure S3 Expression of PTK6 in breast and ovarian cancer cell lines. A) Western blot analysis was performed to assess levels of PTK6 protein expression in a panel of breast cancer cell lines representative of different subtypes, as classified in Neve et al. [54]. B) Western blot analyses were performed to assess levels of PTK6 expression in human ovarian surface epithelial cells and ovarian cancer cell lines.
(0.53 MB TIF)

Figure S4 Effect of IGF-1R kinase inhibitor on IGF-1R signaling. IGF-1R overexpressing MCF-10A cells were treated in suspension cultures containing IGF-1 (100ng/ml) for 48 hours with the indicated concentrations of IGF-1 receptor kinase inhibitor (BMS-536924; Selleck). Lysates were probed with the indicated antisera to assess IGF-1R autophosphorylation and phosphorylation of Akt.
(0.28 MB TIF)

Figure S5 Cluster analysis of chromosome 20 copy number alterations in breast cancers. The heatmap is based on SNP array copy number data from 93 breast tumor samples with 511 chromosome 20 genes (ordered from p arm to q arm). Only data above log2 value 0.5 or below −0.5 were used (representing copy numbers above 2.8 or below 1.4). Left, cluster of the entire chromosome 20; right, magnification of the yellow box. Dark blue, red, and light blue colors represent losses, gains, and no significant change in copy number.
(1.07 MB TIF)

Figure S6 Higher levels of PTK6 mRNA are associated with clinical measures of poor prognosis in multiple breast cancer data sets. Box plots and data are from the Oncomine database (www.oncomine.org). Background corrected expression data are log2 transformed and the median value per microarray is scaled to zero by subtracting the median from each value. The standard deviation of values for each microarray is scaled to 1. P-values are derived from the Student's t-test.
(0.65 MB TIF)
