# Supplementary figures and images for: PTK6 Regulates IGF-1-Induced Anchorage-Independent Survival
Source: PLoS One. 2010 Jul 23;5(7):e11729. doi: 10.1371/journal.pone.0011729 (PMC2909213; doi:10.1371/journal.pone.0011729)

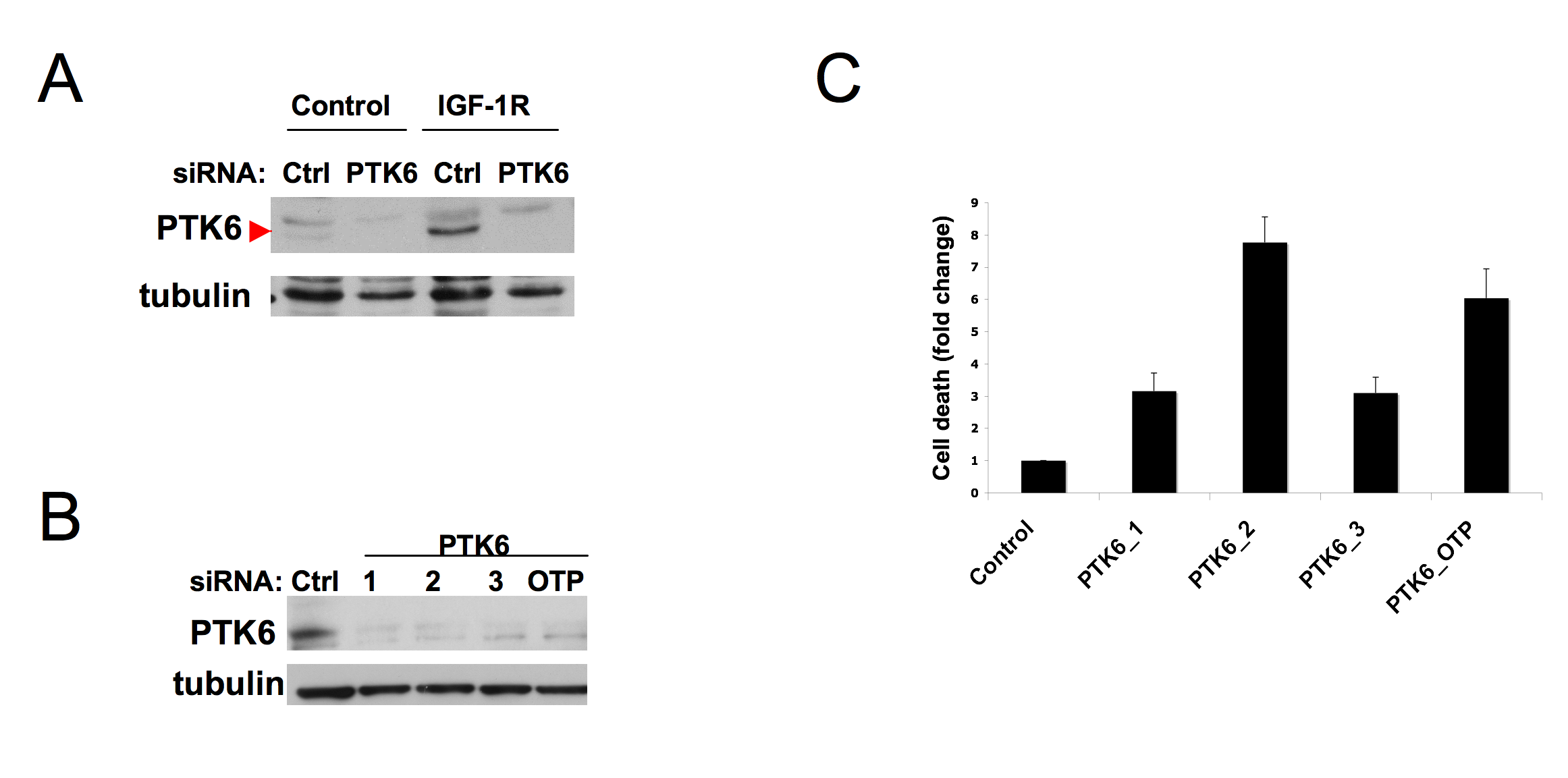

Supplement: Figure S1 — siRNAs targeting PTK6 identified by the screen inhibit anchorage-independent survival of IGF-1R cells. A) Parental and IGF-1R overexpressing MCF-10A cells transfected with control or PTK6 siRNA (OTP, Dharmacon) were cultured in suspension for 48 hours. Lysates were probed with the indicated anti-sera. B) Western analysis demonstrates down regulation of PTK6 expression in IGF-1R cells by siRNAs targeting PTK6 identified by the screen to induce changes in viability. IGF-1R cells transfected with multiple PTK6 siRNAs, including a pool of siRNAs from a distinct vendor (OTP, Dharmacon), were cultured in suspension for 48 hours. Lysates were prepared and probed with the indicated anti-sera. C) siRNAs targeting PTK6 inhibit anchorage-independent survival of IGF-1R cells. IGF-1R cells transfected with multiple PTK6 siRNAs, including a pool of siRNAs from a distinct vendor (OTP, Dharmacon), were cultured in suspension for 48 hours. Cell death was assessed as described in Figure 1. The average fold change from three independent experiments is shown. Error bars indicate standard deviation. (0.24 MB TIF) [file pone.0011729.s001.tif]

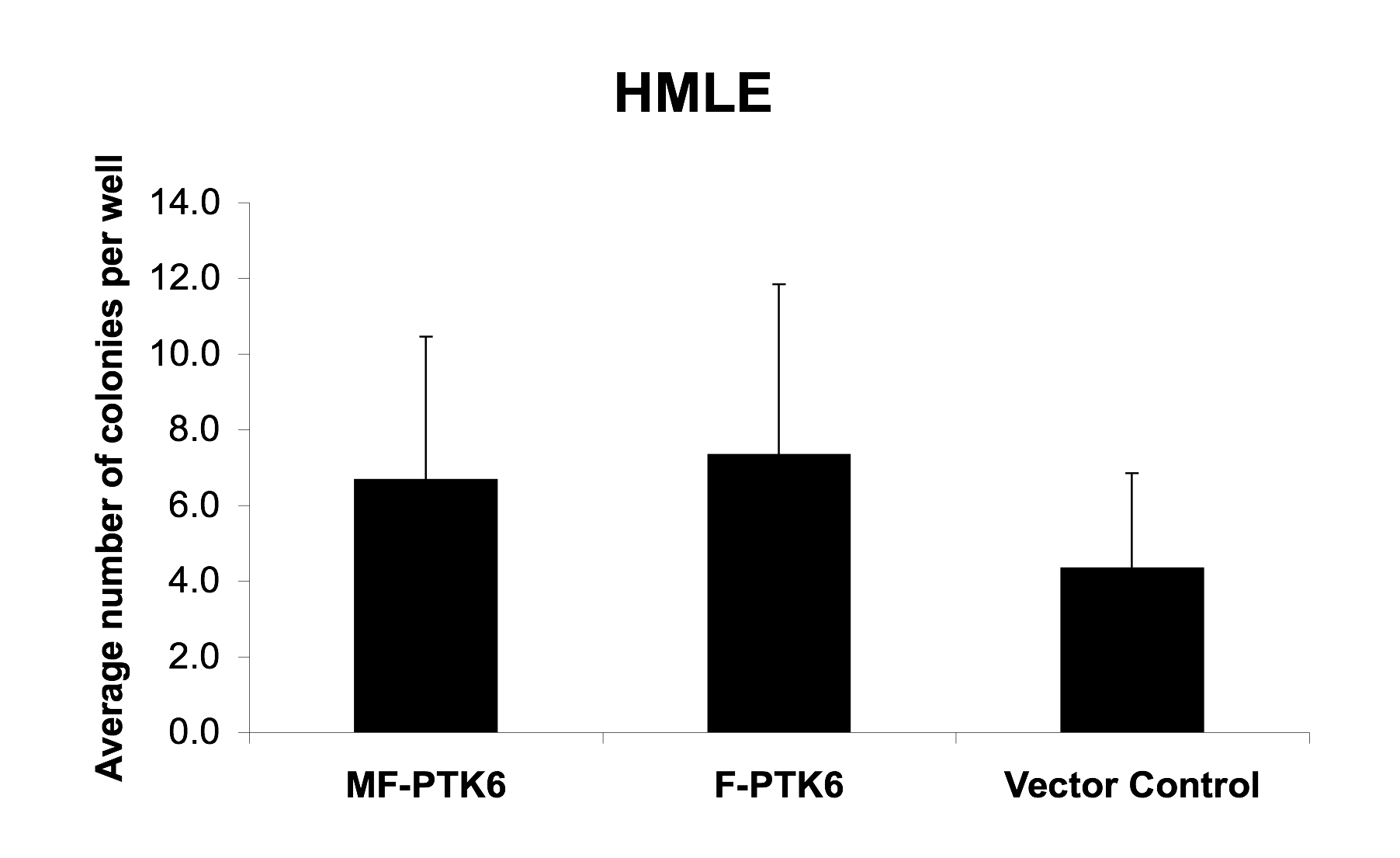

Supplement: Figure S2 — Overexpression of PTK6 alone in HMEC does not enhance growth in soft agar. Immortalized human mammary epithelial cells (HMLE) overexpressing vector control, F-PTK6 or MF-PTK6, were seeded in soft-agar, and colony formation was assessed after four weeks. A representative experiment of three replicate experiments using triplicate wells for each cell line is shown. (0.08 MB TIF) [file pone.0011729.s002.tif]

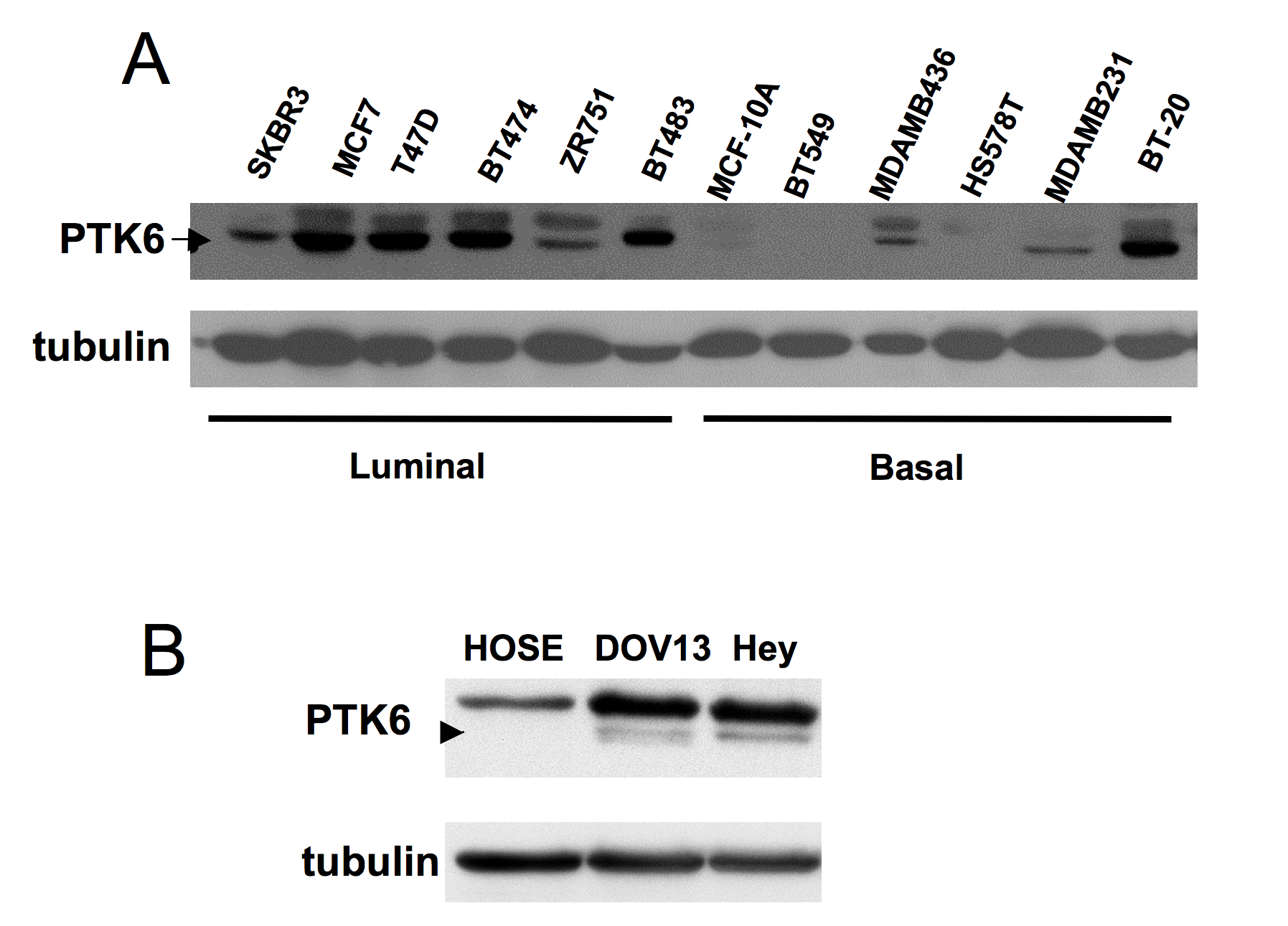

Supplement: Figure S3 — Expression of PTK6 in breast and ovarian cancer cell lines. A) Western blot analysis was performed to assess levels of PTK6 protein expression in a panel of breast cancer cell lines representative of different subtypes, as classified in Neve et al. [54]. B) Western blot analyses were performed to assess levels of PTK6 expression in human ovarian surface epithelial cells and ovarian cancer cell lines. (0.53 MB TIF) [file pone.0011729.s003.tif]

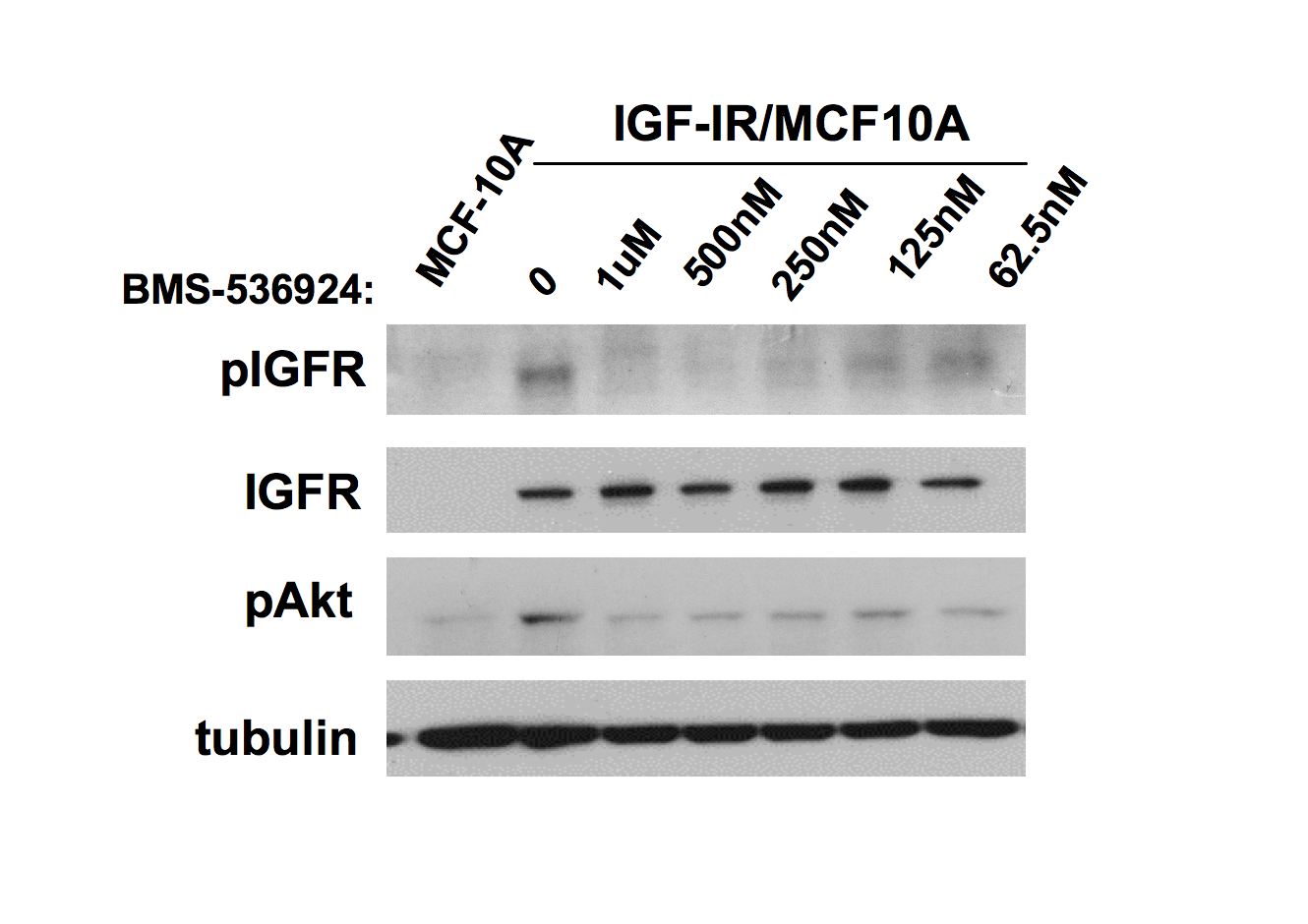

Supplement: Figure S4 — Effect of IGF-1R kinase inhibitor on IGF-1R signaling. IGF-1R overexpressing MCF-10A cells were treated in suspension cultures containing IGF-1 (100ng/ml) for 48 hours with the indicated concentrations of IGF-1 receptor kinase inhibitor (BMS-536924; Selleck). Lysates were probed with the indicated antisera to assess IGF-1R autophosphorylation and phosphorylation of Akt. (0.28 MB TIF) [file pone.0011729.s004.tif]

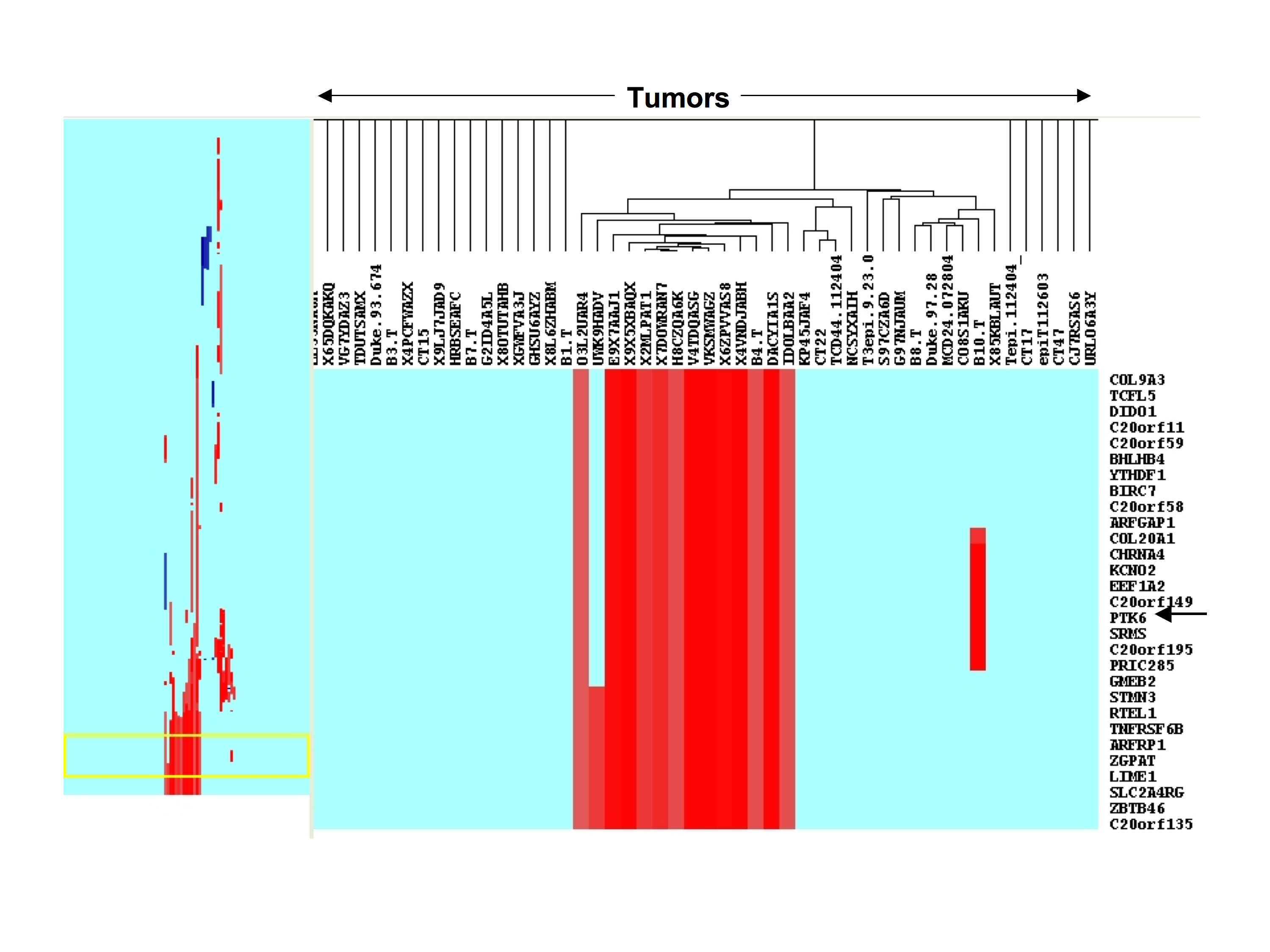

Supplement: Figure S5 — Cluster analysis of chromosome 20 copy number alterations in breast cancers. The heatmap is based on SNP array copy number data from 93 breast tumor samples with 511 chromosome 20 genes (ordered from p arm to q arm). Only data above log2 value 0.5 or below −0.5 were used (representing copy numbers above 2.8 or below 1.4). Left, cluster of the entire chromosome 20; right, magnification of the yellow box. Dark blue, red, and light blue colors represent losses, gains, and no significant change in copy number. (1.07 MB TIF) [file pone.0011729.s005.tif]

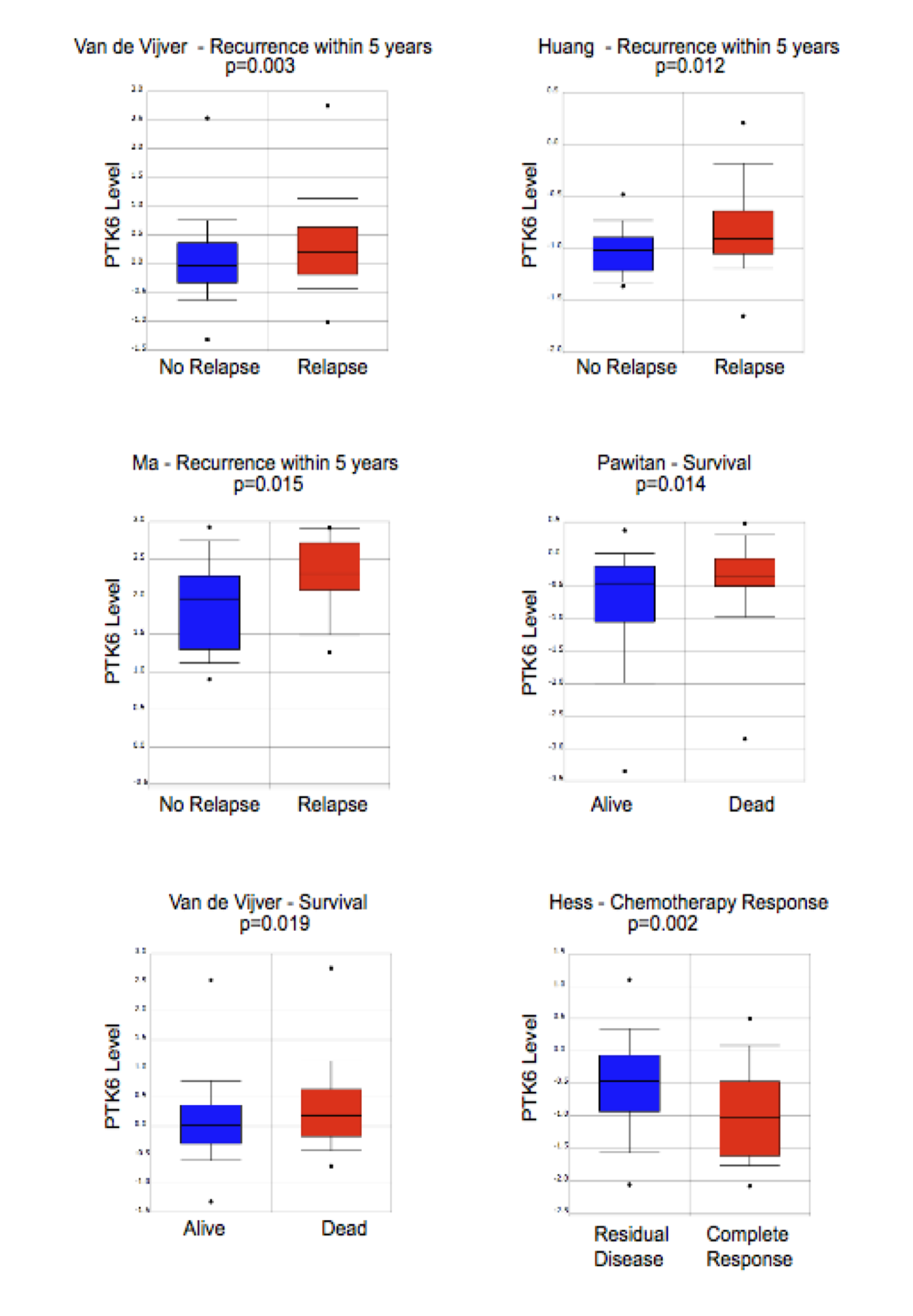

Supplement: Figure S6 — Higher levels of PTK6 mRNA are associated with clinical measures of poor prognosis in multiple breast cancer data sets. Box plots and data are from the Oncomine database (www.oncomine.org). Background corrected expression data are log2 transformed and the median value per microarray is scaled to zero by subtracting the median from each value. The standard deviation of values for each microarray is scaled to 1. P-values are derived from the Student's t-test. (0.65 MB TIF) [file pone.0011729.s006.tif]
